# Neurotensin Modulates the Migratory and Inflammatory Response of Macrophages under Hyperglycemic Conditions

**DOI:** 10.1155/2013/941764

**Published:** 2013-08-13

**Authors:** Liane I. F. Moura, Lucília Silva, Ermelindo C. Leal, Ana Tellechea, Maria Teresa Cruz, Eugénia Carvalho

**Affiliations:** ^1^Center for Neuroscience and Cell Biology, University of Coimbra, 3004-517 Coimbra, Portugal; ^2^CIEPQPF, Chemical Engineering Department, FCTUC, University of Coimbra, 3030-790 Coimbra, Portugal; ^3^Faculty of Pharmacy, University of Coimbra, 3000-548 Coimbra, Portugal; ^4^The Portuguese Diabetes Association (APDP), Rua Do Salitre, No. 118-120, 1250-203 Lisboa, Portugal

## Abstract

Diabetic foot ulcers (DFUs) are characterized by an unsatisfactory inflammatory and migratory response. Skin inflammation involves the participation of many cells and particularly macrophages. Macrophage function can be modulated by neuropeptides; however, little is known regarding the role of neurotensin (NT) as a modulator of macrophages under inflammatory and hyperglycemic conditions. RAW 264.7 cells were maintained at 10/30 mM glucose, stimulated with/without LPS (1 **μ**g/mL), and treated with/without NT(10 nM). The results show that NT did not affect macrophage viability. However, NT reverted the hyperglycemia-induced impair in the migration of macrophages. The expression of IL-6 and IL-1**β** was significantly increased under 10 mM glucose in the presence of NT, while IL-1**β** and IL-12 expression significantly decreased under inflammatory and hyperglycemic conditions. More importantly, high glucose modulates NT and NT receptor expression under normal and inflammatory conditions. These results highlight the effect of NT on cell migration, which is strongly impaired under hyperglycemic conditions, as well as its effect in decreasing the proinflammatory status of macrophages under hyperglycemic and inflammatory conditions. These findings provide new insights into the potential therapeutic role of NT in chronic wounds, such as in DFU, characterized by a deficit in the migratory properties of cells and a chronic proinflammatory status.

## 1. Introduction 

Diabetes mellitus (DM) is characterized by an impaired blood glucose homeostasis, and it affects millions of people in the world [1]. Diabetes can cause poor circulation in the extremities, particularly in people with neuropathy, and long-term diabetes can compromise the immune system increasing the incidence of infections in the patients. One of the most debilitating and costly complications of diabetes is the development of chronic foot ulcers. This disease affects approximately 15% of the diabetic population [2–4]. It can diminish physical activity, and, in extreme cases, diabetic foot ulcerations (DFUs) can lead to lower-limb amputations [5]. Chronic inflammation is a major characteristic of diabetic cutaneous wounds. Wound inflammation has a fundamental role in tissue regeneration [6], while leukocyte dysfunction to the wound site has been shown to contribute to the development of nonhealing wounds [7]. Indeed, diabetic patients show impaired leukocyte function, which has been correlated with hyperglycemia [8]. Studies performed in diabetic patients revealed that normalization of blood glucose levels through insulin administration can improve and ultimately restore the functional activity of neutrophils [9].

It is also well known that an imbalance between proinflammatory and anti-inflammatory cytokines in the diabetic wound tissue compromises the time resolution of inflammation and consequently the healing process [10]. Macrophages play a crucial role in the modulation of the inflammatory response since they can be phenotypically polarized to the classical activated macrophages that stimulate the inflammatory process or to the alternatively activated macrophages that play a role in the resolution of inflammation [11]. Recent results demonstrated that in a diabetic mouse model, impairment in glucose metabolism can cause changes in the macrophage response to lipopolysaccharide (LPS), namely, increased secretion of interleukin 12 (IL-12) and TNF-*α* [12].

In addition to the involvement of inflammation in wound repair responses, various studies suggest that the neuroendocrine system also modulates wound healing [13], specifically through neuropeptides, such as substance P (SP) and neurotensin (NT) [14–18]. NT is a bioactive tridecapeptide that is widely distributed through the brain and the gastrointestinal tract [14, 19]. It regulates a wide range of biological functions, such as the gastric system and inflammatory processes in the lung [14, 18]. Furthermore, NT modulates the immune response, as it interacts with leukocytes, peritoneal mast cells, and dendritic cells, stimulating cytokine release and chemotaxis [20–22]. In particular, neuropeptides, such as NT, are important in modulating macrophage function, due to their direct interaction with macrophages leading to suppression of the production of proinflammatory cytokines and iNOS expression, demonstrating a protective effect in inflammatory conditions [23, 24].

NT mediates its functions through its two G protein coupled receptors: neurotensin receptor 1 (NTR1) and neurotensin receptor 2 (NTR2) (high and low affinity receptors, resp.). A third receptor, the neurotensin receptor 3 (NTR3), is an intracellular, non G protein coupled receptor [5, 25]. Although NT has been implicated in modulating immune responses and macrophage function, its molecular mechanisms of action, under either hyperglycemic or inflammatory conditions or both, remain unclear.

Therefore, this study aims to determine the effect of NT in macrophages function under hyperglycemic and inflammatory conditions.

## 2. Materials and Methods

### 2.1. Materials

LPS from *Escherichia coli* (serotype 026 : B6) was obtained from Sigma Chemical Co. (St. Louis, MO, USA), and NT was obtained from Bachem (Weil am Rhein, Germany). Fetal calf serum was purchased from Invitrogen (Paisley, UK). The protease and phosphatase inhibitor cocktails were obtained from Roche (Mannheim, Germany).

The antibodies against phospho (p), p-p44/42 MAPK, p-p38 MAPK, I*κ*B*α*, and total AKT were purchased from Cell Signaling Technologies (Danvers, MA, USA). The antibodies against pAKT (Ser 473) and the NT receptors were purchased from Santa Cruz Biotechnology (Santa Cruz, California, USA), and the antibodies against total p38 MAPK and p44/42MAPK were purchased from BioLegend (San Diego, CA, USA). The antibody against actin was purchased from Millipore Corporation (Bedford, MA).

All primers were obtained from IDT (Ebersberg, Germany). SYBR green was obtained from Bio-Rad (Hercules, CA, USA), and High Capacity cDNA Reverse Transcription kit was obtained from Applied Biosystems (Carlsbad, CA, USA).

The alkaline phosphatase-linked secondary antibodies and the enhanced chemifluorescence (ECF) reagent were obtained from GE Healthcare (Chalfont St. Giles, UK), and the polyvinylidene difluoride (PVDF) membranes were from Millipore Corporation (Bedford, MA, USA). TRIzol reagent was purchased from Invitrogen (Barcelona, Spain). All other reagents were from Sigma Chemical Co. (St. Louis, MO, USA) or from Merck (Darmstadt, Germany).

### 2.2. Macrophage Cell Culture

RAW 264.7 (mouse leukaemic monocyte macrophage cell line) cells were cultured in DMEM medium, pH 7.4, supplemented with 10% heat inactivated fetal bovine serum (FBS), 3.02 g/L sodium bicarbonate, 100 U/mL penicillin, and 100 *μ*g/mL streptomycin, at 37°C in a humidified incubator containing 5% CO_2_. Subculturing was performed according to ATCC recommendations. The RAW 264.7 cell line was purchased from ATCC (number TIB-71).

### 2.3. Treatments of Macrophages

The cells were incubated in 10 mM (normal glucose) or 30 mM (high glucose) D-glucose, for 15 days, before the beginning of the experiments.

For the viability assay, we used the cells incubated with high glucose for 15 days. Macrophages were treated with 1 *μ*g/mL of LPS, with 10, 50, or 100 nM of NT alone, or with a combination of both treatments (NT plus LPS) for 1, 3, 5, and 7 days. These dose and treatment protocols were selected to evaluate the role of NT in macrophage cell viability after exposure to an acute (1 and 3 days) and a chronic (5 and 7 days) hyperglycemic and inflammatory state.

For the migration assay, RAW 264.7 cells were incubated with 1 *μ*g/mL of LPS alone, with 10 nM of NT alone, or with a combination of both treatments (10 nM NT plus 1 *μ*g/mL LPS) in DMEM medium containing 2% of inactivated fetal calf serum.

In order to study the signal transduction pathways, macrophages were incubated with 1 *μ*g/mL of LPS alone or with 10 nM of NT alone or a combination of both treatments (10 nM NT plus 1 *μ*g/mL LPS) for 5, 15, 30, or 60 min. The cells were incubated with the same treatments for 24 h to evaluate the levels of NT receptors and for 6 h in the real-time PCR studies.

### 2.4. MTT Viability Assay

RAW 264.7 (8 × 10^4^ cells/well) cells were plated in 48-well plates in 430 *μ*L of DMEM. After cell treatment, as described previously, 43 *μ*L of MTT solution (5 mg/mL) were added to each well. The plates were further incubated at 37°C for 1 h in a humidified incubator containing 5% CO_2_. 300 *μ*L of acidic isopropanol (0.04 NHCl in isopropanol) were then added to each well and mixed in order to dissolve the dark blue crystals of formazan. Acidic isopropanol was collected to an ELISA microplate, and formazan quantification was performed using an ELISA automatic microplate reader (SLT, Austria) at 570 nm, with a reference wavelength of 620 nm.

### 2.5. *In Vitro* Scratch Migration Assay

RAW 264.7 (4 × 10^5^ cells/well) cells were resuspended in 3 mL of DMEM medium in *μ*-Dish^35mm, high^ (ibidi). After 24 h, a “scratch” was made, with a pipette tip, in the cell monolayer in a straight line to create an area without cells. The medium was removed, and cells were washed two times with PBS. DMEM medium containing 2% of inactivated fetal calf serum was then added to the cells to diminish cell proliferation. The cells were incubated as described previously and allowed to migrate for 24 h. Photographs were captured with a coupled AxioCam MR3 camera with PALM reflector and 5x objective, using an inverted Axiovert 200. A specific numbered/lettered area was chased to permit later recognition of the photographed area. After the incubation period, photographs were taken in the same area where the first photograph was taken. Photographs were analysed, and the number of cells in the scratch area was counted. For the analysis, the number of cells in the zero point was taken into account.

### 2.6. Western Blotting

RAW 264.7 (1.5 × 10^6^ cells/well) cells were seeded in 6-well plates and treated as described before. Cells were then washed twice with ice-cold PBS buffer and lysed with RIPA buffer (50 mM Tris HCl pH 8, 150 mM NaCl, 1% NP-40 (Nonidet P-40), 0.5% Sodium Deoxycholate, 0.1% SDS, 2 mM EDTA, protease inhibitor cocktail, phosphatase inhibitor cocktail, and 1 mM DTT). Protein concentration was determined using the bicinchoninic acid method, and cell lysates were denatured at 95°C, for 5 min, in sample buffer (0.125 mM Tris pH 6.8; 2% w/v SDS; 100 mM DTT; 10% glycerol; and bromophenol blue) for its use in western blot analysis. 30 *μ*g of total protein were resolved on 10% SDS-PAGE and transferred to PVDF membranes. The membranes were blocked with 5% (w/v) fat-free dry milk in Tris-buffered saline containing 0.1% (v/v) Tween 20 (TBS-T), for 1 h, at room temperature. After blocking and washing, membranes were incubated overnight at 4°C with the primary antibodies against the different proteins of interest: p-p38 MAPK (1 : 1000), I*κ*B-*α* (1 : 1000); p-p44/42MAPK (1 : 1000), p-AKT (1 : 500), NTR1 (1 : 500), NTR2 (1 : 500), or NTR3 (1 : 500). After incubation, membranes were washed and incubated for 1 h at room temperature with alkaline phosphatase-conjugated antirabbit antibody (1 : 5000) or alkaline phosphatase-conjugated antimouse antibody (1 : 5000). The membranes were exposed to ECF reagent followed by scanning for blue excited fluorescence on the VersaDoc (Bio-Rad Laboratories, Amadora, Portugal). To test whether similar amounts of protein for each sample were loaded, the membranes were stripped and reprobed with antibodies for total, p38 MAPK, p44/42MAPK, and AKT or with an antiactin antibody, and blots were developed with alkaline phosphatase-conjugated secondary antibodies and visualized by enhanced chemifluorescence. The generated signals were analyzed using the Image-Quant TL software.

### 2.7. Real-Time RT-PCR

Cells (2 × 10^6^ cells/well) were seeded in 6-well plates and treated as described before. Total RNA was isolated from cells with the TRIzol reagent according to the manufacturer's instructions, and concentration was determined by OD260 measurement using the NanoDrop spectrophotometer (Thermo Scientific, USA). First strand cDNA was synthesized using High Capacity cDNA Reverse Transcription. Briefly, 2 *μ*L of 10x RT Buffer, 0.8 *μ*L of 25x dNTP Mix, 2 *μ*L of 10x RT random primers, 1 *μ*L of MultiScribe Reverse Transcriptase, and 4.2 *μ*L of nuclease free H_2_O were added to 10 *μ*L of RNA (1 *μ*g) sample. Then, real-time RT-PCR was performed in a Bio-Rad iCycler iQ5. For each reaction, 10 *μ*L volume were used containing 2.5 *μ*L cDNA, 5 *μ*L 2x SYBR Green Supermix, 1 *μ*L of each primer (250 nM), and 0.5 *μ*L of H_2_O PCR grade. Primer sequences are given in Table 1. Gene expression changes were analyzed using iQ5 optical system software v2. The software enables analysis of the results with the Pfaffl method [26]. The results were normalized using a reference gene, hypoxanthine phosphoribosyltransferase 1 (HPRT-1), that was selected based on our previous results demonstrating that it does not change under these conditions.

### 2.8. Statistical Analysis

Results are expressed as mean ± SEM. Statistical analysis was performed using one-way ANOVA followed by Tukey's multiple comparison tests or through the unpaired Student's *t* test using GraphPad Prism (GraphPad Software Inc., San Diego, CA, USA). *P* values less than 0.05 were considered statistically significant.

## 3. Results

All experiments were performed using RAW 267.4 cells incubated with either 10 mM glucose (normal glucose) or 30 mM glucose (high glucose), for a period of 15 days.

### 3.1. Cell Viability under Hyperglycemic Conditions

**Figure 1:**
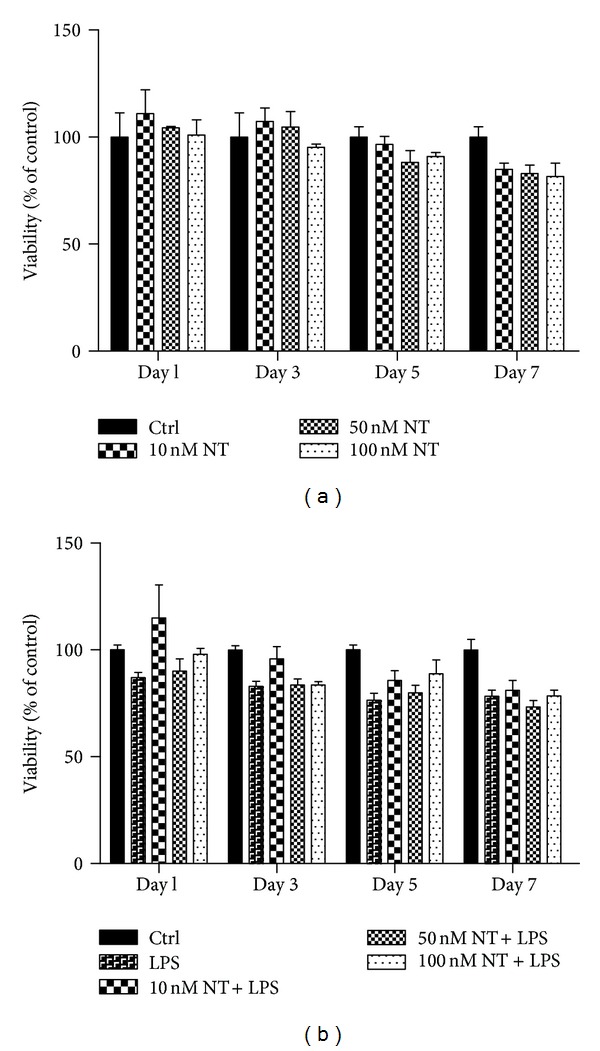
Viability of macrophages, under either 10 or 30 mM glucose, by MTT assay. RAW 264.7 cells were plated at 8 × 10^4^/well and were treated with 10, 50 or, 100 nM of NT (a) or in combination with 1 *μ*g/mL of LPS (b) for 7days. After, 1, 3, 5, or 7 days of incubation, MTT assay was performed as described in “Materials and Methods.” Absorbance quantification was performed using a microplate reader at 570 nm, with a reference wavelength of 620 nm. Results are presented as mean ± SEM of three independent experiments.

NT treatment did not change the viability of macrophages under hyperglycemic conditions either in the absence or presence of LPS (Figures 1(a) and 1(b), resp.). Since no major differences were observed between the different doses of NT used (10, 50, or 100 nM), the following experiments were performed using 10 nM of NT.

### 3.2. Migration of Macrophages under Normal or Hyperglycemic Conditions

**Figure 2:**
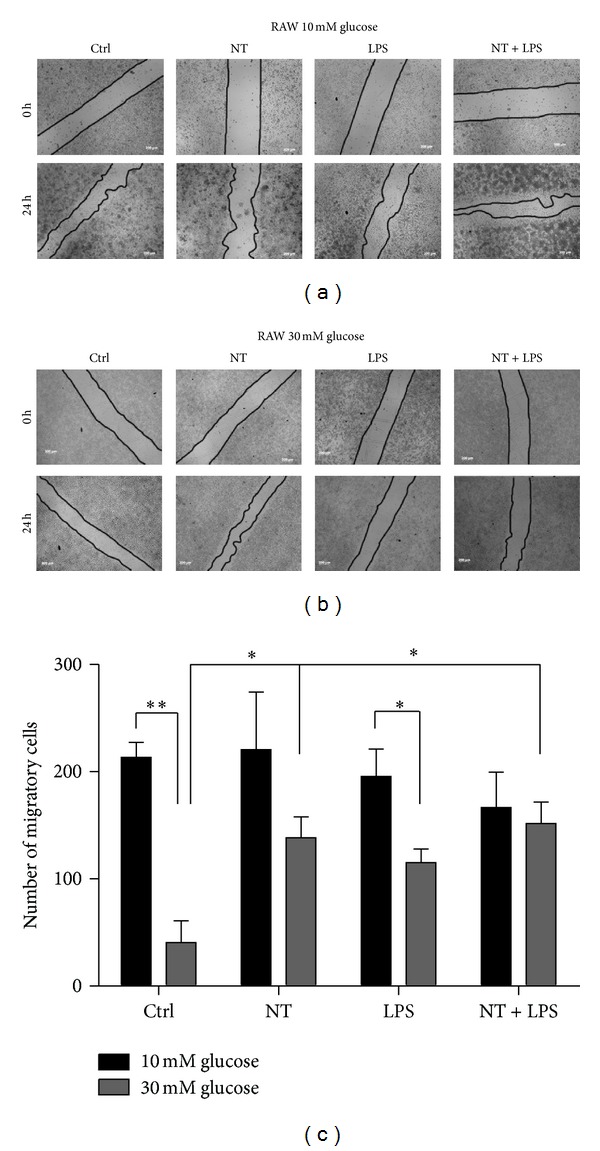
Migration of macrophages at 10 (a) and 30 mM (b) glucose, by *in vitro* scratch assay. (c): number of migrating cells (referred before). Cells were plated at 4 × 10^5^/well and treated with 10 nM NT or 1 *μ*M/mL LPS or both, during 24 h. The scratch assay was performed as described in “Material and Methods.” The images were acquired by transmission microscopy, and photographs were taken before cell treatment (0 h) and 24 h after treatments. Magnification used was 40x. Results are presented as mean ± SEM of three independent experiments. *P < 0.05; **P < 0.01.

Our results show that under normoglycemic conditions (10 mM glucose), NT treatment did not stimulate macrophage migration, either in the absence or in the presence of LPS (Figures 2(a) and 2(c)). However, under hyperglycemic conditions (30 mM glucose), NT significantly increased cell migration compared to control (*P* < 0.05) as shown in Figures 2(b) and 2(c). Moreover, high glucose alone (*P* < 0.01) or in combination with LPS treatment (*P* < 0.05) significantly decreased macrophage migration when compared with normoglycemic conditions (Figures 2(b) and 2(c)).

These results demonstrate that macrophage migration is impaired under hyperglycemic conditions. Moreover, this impairment is partially reverted by NT treatment.

### 3.3. Inflammatory Cytokine Expression under Normal and Hyperglycemic Conditions

**Figure 3:**
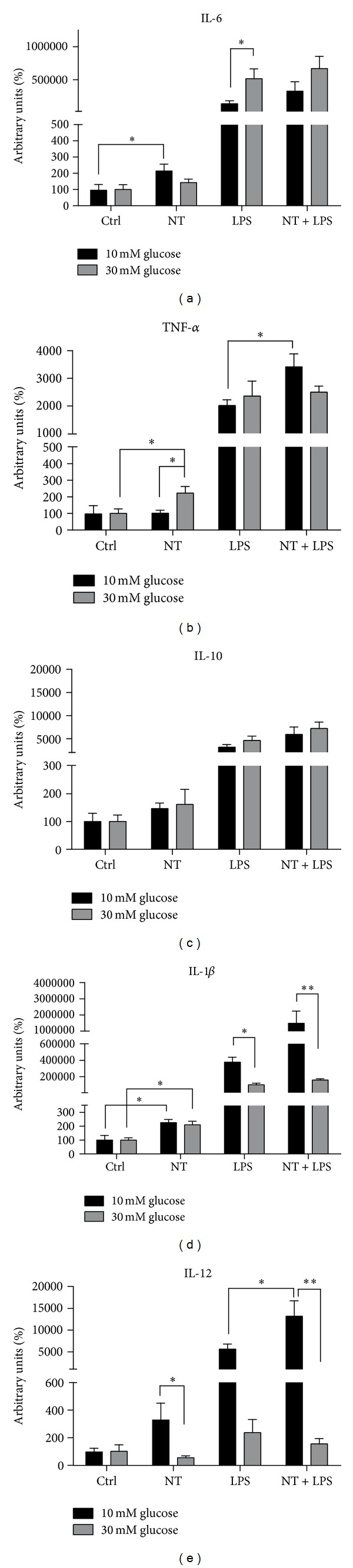
Expression of inflammatory cytokines IL-6, TNF-*α*, IL-10, IL-1*β*, and IL-12 in macrophages, at 10 and 30 mM glucose, by real-time PCR. Cells were plated at 2 × 10^6^/well and treated with 10 nM NT or 1 *μ*g/mL LPS or both, during 6 h. Total RNA was isolated as described in “Materials and Methods.” The relative gene expression is indicated as arbitrary units and was obtained after normalization with the HPRT gene. Results are presented as mean ± SEM of six to ten independent experiments. *P < 0.05; **P < 0.01.

In order to address the pattern of cytokine gene expression that is involved in wound healing processes stimulated by NT alone or in the presence of LPS, we measured gene expression for the proinflammatory cytokines IL-6, TNF-*α*, IL-1*β*, and IL-12 and for the anti-inflammatory cytokine IL-10 in macrophages, as indicated in Figure 3.

Under 10 mM glucose, NT induced a significant overexpression of IL-6 (*P* < 0.05) and IL-1*β* (*P* < 0.05). On the other hand, under 30 mM glucose, NT significantly increased the expression of TNF-*α* (*P* < 0.05) and IL-1*β* (*P* < 0.05), as compared to high glucose alone (Figure 3).

Moreover, in LPS-treated cells, NT treatment significantly increased TNF-*α* (*P* < 0.05) and IL-12 (*P* < 0.05) expression under 10 mM glucose, when compared with LPS-treated cells (Figure 3). However, NT did not alter the expression of these genes under hyperglycemic conditions. Interestingly, hyperglycemia alone increased the expression of IL-6 (*P* < 0.05) and decreased the expression of IL-1*β* (*P* < 0.05), when compared to normal glycemia under inflammatory conditions (Figure 3). Overall, these results show that NT modulates the inflammatory profile of macrophages; however, this effect was not observed under hyperglycemic conditions, as observed in Figure 3.

### 3.4. Modulation of Intracellular Signaling Pathways by NT in LPS Treated Macrophages under Either 10 Or 30 mM Glucose

**Figure 4:**
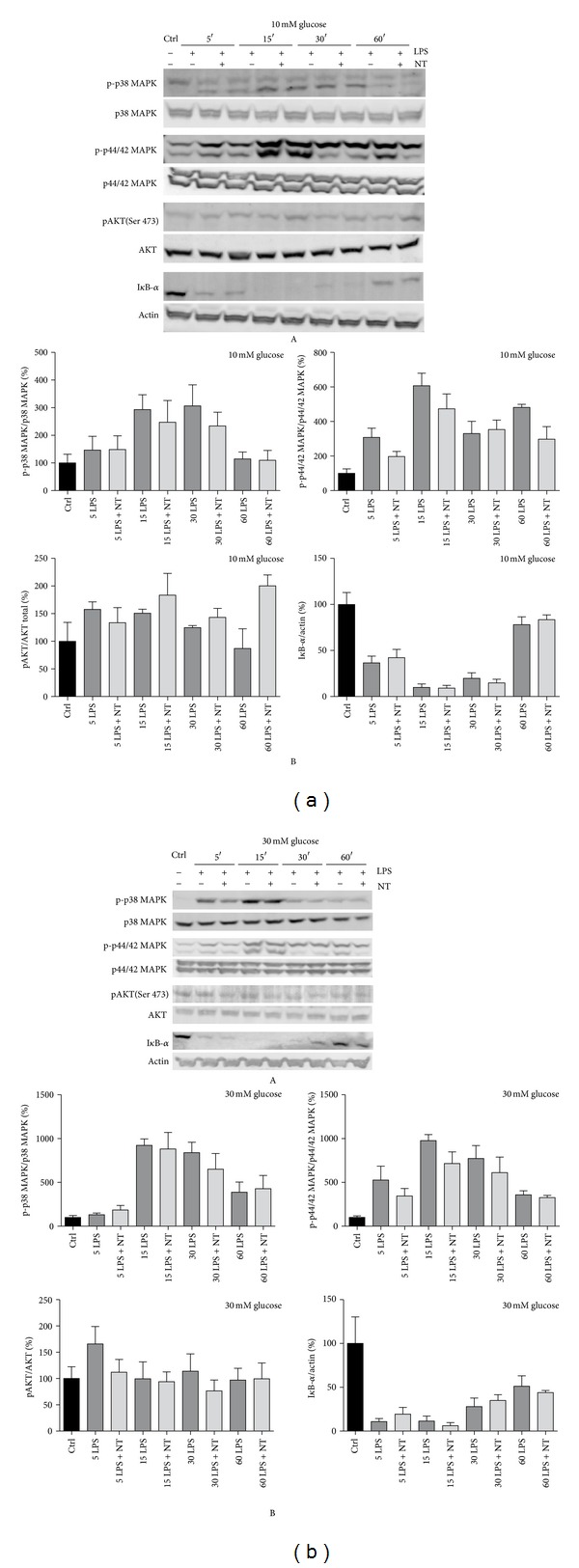
Modulation of LPS activated signaling pathways by NT at 10 (a) and 30 mM glucose (b), in macrophages, by western blot (A) and relative quantification (B). Cells were plated at 1.5 × 10^6^/well and treated simultaneously with 10 nM NT and 1 *μ*g/mL LPS during 5, 15, 30, or 60 minutes. The lysates were probed for phospho p38 MAPK, phospho p44/42 MAPK, phospho pAKT (Ser 437), and inhibitory protein for NF-*κ*B activation, I*κ*B-*α* antibodies. Equal amounts of protein were evaluated with total p38 MAPK, p44/42 MAPK, AKT, and actin antibodies. The results shown are representative of four to six independent experiments with similar results.

The expression of proinflammatory molecules is tightly regulated by several transcription factors and signaling pathways. Among these pathways, mitogen-activated protein kinases (MAPKs) and the transcription factor NF-*κ*B constitute signaling molecules that play critical roles in the orchestration of an inflammatory response. The effect of NT on LPS-induced molecular pathway activation, under either 10 or 30 mM glucose, was assessed by measuring the levels of the phosphorylated forms of MAP kinases (p38 MAPK, p44/42 MAPK, and SAPK/JNK) and PKB/AKT by western blot. The importance of the transcription factor NF-*κ*B was also evaluated by determination of the protein levels of its inhibitory protein, I*κ*B-*α*, as shown in Figures 4(a) and 4(b). No significant differences were observed after NT treatment in the presence of LPS, in the presence of either 10 or 30 mM glucose, as compared to cells treated with LPS alone. 

### 3.5. Modulation of NT Receptors under Normal and Hyperglycemic Conditions

**Figure 5:**
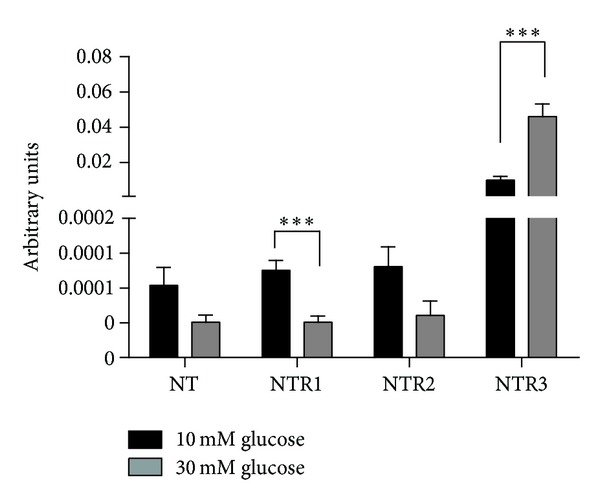
Expression of neurotensin and neurotensin receptors, NTR1, NTR2, and NTR3 in macrophages at 10 and 30 mM glucose, by real-time PCR. Cells were plated at 2 × 10^6^/well and maintained at the indicated conditions. Total RNA was isolated as described in “Materials and Methods.” The relative gene expression is indicated as arbitrary units and was obtained after normalization with the HPRT gene. Results are presented as mean ± SEM of five to eight independent experiments. ***P < 0.001.

**Figure 6:**
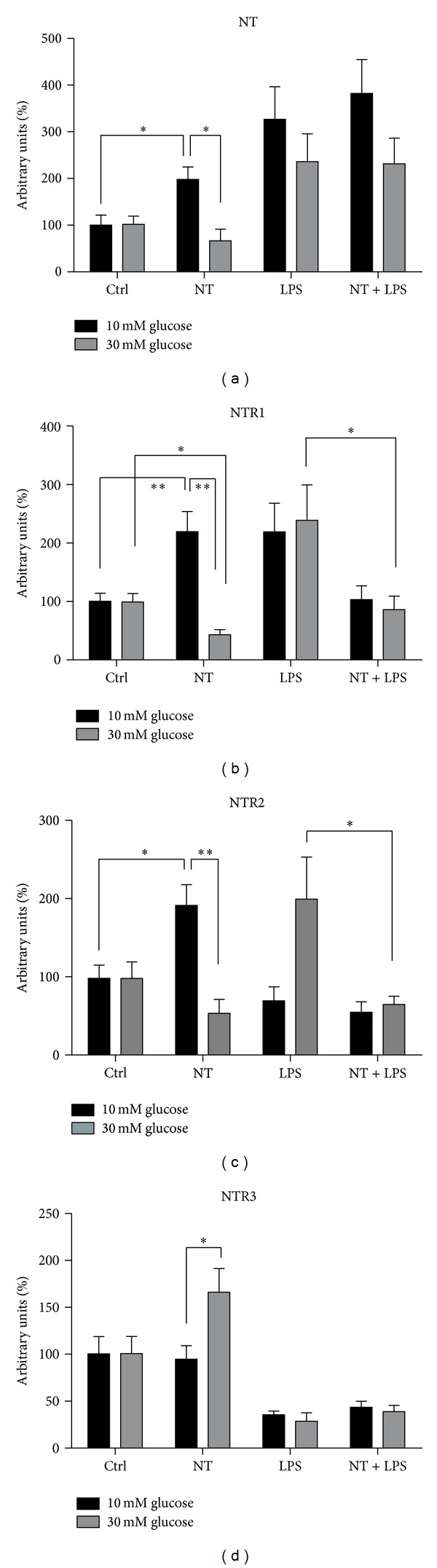
Expression of neurotensin and neurotensin receptors, NTR1, NTR2, and NTR3 in macrophages at 10 and 30 mM glucose, by real-time PCR. Cells were plated at 2 × 10^6^/well and treated with 10 nM NT or 1 *μ*M/mL LPS or both, during 6 h. Total RNA was isolated as described in “Materials and Methods.” The relative gene expression is indicated as arbitrary units and was obtained after normalization with the HPRT gene. Results are presented as mean ± SEM of six to nine independent experiments. *P < 0.05; **P < 0.01.

Gene expression results showed that under hyperglycemic conditions, NTR1 was significantly decreased (*P* < 0.001), while both NTR2 and endogenous NT were not changed, compared to normal glycemia in these cells. Interestingly, the expression of NTR3 was significantly increased under hyperglycemic conditions (*P* < 0.001) when compared to normal glycemia. In addition, the NTR3 was the most expressed receptor in macrophages under either 10 or 30 mM glucose, as shown in Figure 5. Furthermore, we also evaluated how NT, LPS, or the cotreatment of macrophages with both agents affect the expression of endogenous NT and its receptors (Figure 6). The endogenous NT gene expression is significantly increased under NT-treated cells at 10 mM glucose (*P* < 0.05), when compared to nontreated cells. This effect does not occur when the cells were incubated under hyperglycemia.

In addition, NT-treated cells significantly increased (*P* < 0.01) NTR1 expression under 10 mM glucose, whereas in cells maintained under 30 mM glucose, NTR1 expression was significantly decreased (*P* < 0.05). Furthermore, in LPS-treated cells, NTR1 expression was significantly increased when compared to untreated cells, both under 10 and 30 mM of glucose. However, NT significantly decreased NTR1 expression in 30 mM glucose (*P* < 0.05) (Figure 6). A similar pattern of expression was observed for NTR2. In NT-treated cells, NTR2 expression was increased (*P* < 0.05) when compared to untreated cells in 10 mM glucose but not in hyperglycemic condition, similar to what was previously observed for both NTR1 and endogenous NT expression. In the presence of LPS, NTR2 expression was significantly increased in 30 mM glucose (*P* < 0.05), and this effect was not observed in NT-treated cells. Moreover, hyperglycemia did not change NTR3 expression but in the presence of NT, NTR3 expression was significantly increased (Figure 6). However, under inflammatory conditions, NTR3 gene expression was decreased compared to untreated cells, and no further changes were observed in the presence of NT, as shown in Figure 6. Interestingly, after macrophage treatment with exogenous NT, the expression of endogenous NT and its two extracellular receptors, NTR1 and NTR2 was significantly increased compared to untreated cells under 10 mM glucose. However, this effect of NT treatment was not found in hyperglycemic condition. The opposite effect was observed for the intracellular receptor, NTR3, where hyperglycemia significantly increased NTR3 expression, but not in the presence of exogenous NT.

**Figure 7:**
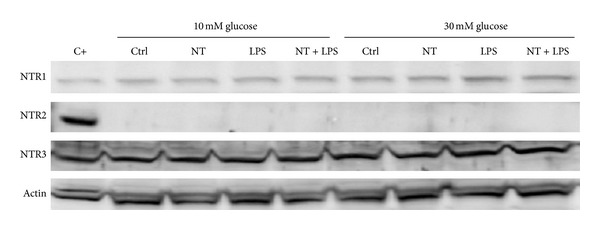
Neurotensin receptor protein levels in macrophages at 10 and 30 mM glucose by western blot. Cerebral cortex lysate (C+) was used as the positive control. RAW 264.7 cells were plated at 1.5 × 10^6^/well and maintained at the indicated conditions. The lysates were probed for NTR1, NTR2, and NTR3 and actin antibodies. Three independent experiments were done for each antibody.

At the protein level, however, no differences were observed in NTR1 or NTR3 levels in 10 mM or 30 mM glucose either in the presence or absence of LPS (Figure 7), while NTR2 was undetectable in these cells, as we have shown previously [22].

## 4. Discussion 

Platelets, neutrophils, fibroblasts, and macrophages contribute to wound healing by releasing cytokines, interleukins, and growth factors. These important cellular mediators modulate the inflammatory phase of healing [2, 8]. Macrophages, in particular, play an important role in inflammatory and immune processes. Physiological and pathophysiological events can be activated and ultimately regulated by neuropeptides, such as SP and/or NT [18, 27, 28]. It is known that local acute inflammation and migration are crucial events for proper wound healing and that chronic low-grade inflammation contributes to the impaired healing observed in diabetes [4, 5].

Our results demonstrate that, under hyperglycemia, NT decreases the inflammatory response of macrophages and stimulates their capacity of migration. This is, to the best of our knowledge, the first study that evaluates the effect of NT in macrophages under either inflammatory or hyperglycemic conditions or both. These findings highlight the potential therapeutic role of NT in compromised wound healing conditions, such as diabetic foot ulcers, characterized by a pathological proinflammatory status and impaired cell migration. Accordingly, in an *in vitro* cerebral wound healing model, NT was shown to play an important role in response to inflammation or lesions in the central nervous system through the NTR3 [29]. Moreover, Brun et al. 2005 [14] verified that NT, through NT receptor 1, stimulates epithelial restitution in intestine mucosa through a COX-2 dependent pathway in chronic inflammation of the intestine.

In addition, we observed a reduction in the macrophage migratory profile under hyperglycemic conditions, when compared to normal glycaemia. However, NT was able to highly improve the migratory capacity of these cells, either under normal or inflammatory conditions. Accordingly, Martin et al. 2003 [29] observed that NT stimulates migration of a human microglial cell line C13NJ in normoglycemic conditions. Furthermore, NT significantly stimulates the phagocytic process of peritoneal macrophages from BALB/c mice [30]. Moreover, these results show that NT increases the migratory capacity of macrophages but not cell proliferation, since the MTT assay did not show any proliferative differences either in the presence or in the absence of NT. These results suggest that NT stimulates the migratory response of macrophages in the diabetic state.

The pattern of inflammatory cytokines expressed by macrophages is affected under high glucose conditions. It is known that diabetes induces the expression of various cytokines, such as TNF-*α* and IL-6, by immune cells [31–33]. IL-6 is secreted by T cells and macrophages and acts as a pro-inflammatory cytokine to stimulate the immune response [34]. TNF-*α* is one of the major inflammatory mediators secreted by macrophages upon a proinflammatory stimulation and is expressed constitutively at a low level in monocytic cells. This basal level expression of TNF-*α* has been shown to be altered by the inflammatory milieu leading to either its upregulation or downregulation [35]. Particularly, TNF-*α* gene expression is increased in the presence of NT in 30 mM glucose but not in 10 mM glucose. However, in the presence of LPS, TNF-*α* expression is increased, and this effect was even more pronounced in the presence of NT, as observed in 10 mM glucose, but not in 30 mM glucose. IL-1*β* is produced by activated macrophages and is an important mediator of the inflammatory response; it is involved in a variety of cellular activities including cell proliferation, differentiation, and apoptosis [34]. IL-12, a cytokine produced mainly by monocytes/macrophages, is a central inducer of cell-mediated immunity that promotes the development, proliferation, and function of T helper 1 (Th1) cells [35]. Specifically, IL-1*β* and IL-12 gene expression was markedly decreased when the cells were treated with NT and LPS in 30 mM glucose. Hill et al. [36] demonstrated that hyperglycemia inhibits IL-1 release from LPS-activated macrophages, a key mediator of the immune response against infection. Thus, different glucose concentrations can change the phenotype of macrophages leading to a switch from a proinflammatory to an anti-inflammatory profile after cell treatment with normal or high glucose concentration, respectively, as observed in other cells such as lymphocytes. This imbalance in the Th1/Th2 homeostasis contributes to the onset and progression of diabetes [37]. This may justify the prevalence of infections in poorly controlled diabetics. Our results show that NT inhibits the inflammatory response of macrophages under hyperglycemic conditions. NT induces cytokine/chemokine expression, such as macrophage inflammatory protein (MIP)-2, monocyte chemotactic protein (MCP)-1, IL-1*β*, and TNF-*α* through p44/42MAPK and PI-3 K-associated pathways in a murine microglial cell line [38]. We, on the other hand, have demonstrated that NT does not activate p38 MAPK, p44/42MAPK, and PKB/AKT signaling pathways under either 10 mM or 30 mM glucose. More importantly, we observed a significant decrease in endogenous NT and NTR expression in hyperglycemic conditions which correlates with the high glucose-induced decrease in macrophage migration. Understanding which of these receptors might be involved in the inflammatory response of macrophages induced by NT will be important in order to better delineate the mechanisms involved in the effects of NT.

Our results demonstrate that the NTR3 was the most expressed receptor in macrophages. Similar results were obtained by Martin et al. 2003 [29] in a human microglial cell line. A downregulation of the VPAC2 (receptor for vasoactive intestinal peptide) expression has also been shown after 4 weeks of diabetes, as observed by Dvoráková et al. 2006 [39], indicating that hyperglycemia may impair signal transduction through these receptors.

Furthermore, under 10 mM glucose, NT significantly stimulated endogenous NT, NTR1, and NTR2 expression, while no changes were observed for NTR3. On the other hand, in cells under 30 mM glucose, NT highly increased NTR3 expression. However, NT and NTR2 protein expression was not detected in these cells (data not shown for NT). Similar results were observed previously by da Silva et al. 2011 [22], where the NTR3 was the most expressed receptor in a dendritic cell line under normoglycemic conditions. These differences in the protein expression of NT receptors could be due to the NTR3 localization, since it is an intracellular receptor, and its responses can be mostly mediated by endogenous NT. These results suggest that hyperglycemia causes the decrease in the levels of cell surface receptors, increasing the number of receptors in the light vesicle fraction without changes in the binding affinity for the peptide, and consequently, internalization of receptor 3 [40, 41]. Furthermore, under inflammatory conditions, endogenous NT is highly expressed in either the presence or absence of exogenous NT, while NTR1 and NTR2 are greatly expressed in the presence of LPS, but when NT is present, their expression returns to control levels. On the other hand, the expression of NTR3 is decreased compared to noninflammatory conditions. It is known that in peripheral tissues, such as gastrointestinal tract, desensitization of NT receptors to NT seems to be frequent [40, 42]. Furthermore, in hyperglycemia, the loss of G protein-coupled receptor function is mainly caused by reduced affinity for the neurotensin [43]. These results indicate that the effect of NT is masked by high glucose and/or reduction of the NT affinity to the receptors, as observed for other neuropeptides in similar conditions [44]. Further studies to better understand the role of NT receptors in inflammatory and hyperglycemic conditions are needed.

Furthermore, and in agreement with our results, Matyal et al. 2011 [45] observed that in diabetic patients, atrial cardiac tissue neuropeptide Y expression is decreased and its receptors Y2 and Y5 mRNA levels are upregulated. Altered expression of neuropeptide Y and its receptors during hyperglycemia may contribute to coronary artery disease, due to decreased angiogenesis, increased apoptosis, and increased vascular smooth muscle proliferation. Under these conditions, NT promotes an earlier acute inflammatory response reflecting possible beneficial effects for diabetic wound healing.

## 5. Conclusion

These studies demonstrate that NT affects macrophage responses, both under inflammatory and hyperglycemic conditions, through the stimulation of cell migration and regulation of cytokine expression. These *in vitro* results are the starting point to find relevant molecules and signaling pathways triggered by NT under inflammatory and hyperglycemic conditions that are currently being confirmed both in the *in vivo* models as well as in the primary macrophage cultures. Based on the present results obtained, we suggest that NT administration under normal glucose conditions promotes an inflammatory response by macrophages, which may be important in the early phases of healing. When administered under hyperglycemic conditions, NT stimulates migration but inhibits the proinflammatory status of macrophages thus contributing to the resolution of inflammation and allowing the progression to the migration-remodeling phases of wound healing. These effects have the potential to be beneficial in a diabetic wound environment.

## Figures and Tables

**Table 1:** Forward and reverse primers sequences used in RT-PCR.

Primer	5′-3′ sequence (forward; reverse)
NT	For: AATGTTTGCAGCCTCATAAATAAC
	Rev: TGCCAACAAGGTCGTCATC
NTR1	For: GGCAATTCCTCAGAATCCATCC
	Rev: ATACAGCGGTCACCAGCAC
NTR2	For: GCCATTACTAACAGTCTAAGC
	Rev: GCAATTCGTCCTATTCTACAC
NTR3	For: ATGGCACAACTTCCTTCTG
	Rev: AGAGACTTGGAGTAGACAATG
IL-6	For: TTCCATCCAGTTGCCTTC
	Rev: TTCTCATTTCCACGATTTCC
TNF-*α*	For: CAAGGGACTAGCCAGGAG
	Rev: TGCCTCTTCTGCCAGTTC
Il-10	For: CCCTTTGCTATGGTGTCCTTTC
	Rev: ATCTCCCTGGTTTCTCTTCCC
IL-1*β*	For: ACCTGTCCTGTGTAATGAAAG
	Rev: GCTTGTGCTCTGCTTGTG
IL-12	For: CAGAAGCTAACCATCTCCTGGTTTG
	Rev: TCGGGAGTAATTTGGTGCTTCACAC
HPRT1	For: GTTGAAGATATAATTGACACTG
	Rev: GGCATATCCAACAACAAAC
